# Correlation of obesity, dietary patterns, and blood pressure with uric acid: data from the NHANES 2017–2018

**DOI:** 10.1186/s12902-022-01112-5

**Published:** 2022-08-05

**Authors:** Jia Yao, Yuan Zhang, Jia Zhao, Yu-Ping Lin, Qi-Yun Lu, Guan-Jie Fan

**Affiliations:** 1 School of Second Clinical Medicine, Guangzhou University of Chinese Medicine, Guangzhou, 510000 China; 2 Department of Endocrinology, The Second Affiliated Hospital of Guangzhou University of Chinese Medicine, Guangzhou, 510000 China; 3 Department of Endocrinology, Guangdong Provincial Hospital of Chinese Medicine, 111 Dade Road, Yuexiu District, Guangzhou, 510120 China

**Keywords:** Uric acid, Obesity, Diet, Blood pressure, NHANES

## Abstract

**Background:**

Prevalence rates of hyperuricemia and gout are increasing. Clinical investigations of hyperuricemia-related risk factors aid in the early detection, prevention, and management of hyperuricemia and gout. Ongoing research is examining the association of obesity, dietary patterns, and blood pressure (BP) with serum uric acid (sUA).

**Methods:**

A cross-sectional study was conducted based on the National Health and Nutrition Examination Survey. The exposures included body mass index (BMI), dietary patterns, and BP. The outcome variable was sUA level. The weighted multivariate linear regression models and smooth curve fittings were used to assess the association of BMI, dietary patterns, and BP with sUA.

**Results:**

There was a significantly positive correlation between BMI and sUA (β = 0.059, 95% CI: 0.054 to 0.064, *P* < 0.00001). Overweight and obese individuals had higher sUA levels than those with the normal BMI (β = 0.451, 95% CI: 0.357 to 0.546, *P* < 0.00001; β = 0.853, 95% CI: 0.760 to 0.946, P < 0.00001; respectively). Dietary energy intake was positively correlated with sUA (β = 0.000, 95% CI: 0.000 to 0.000, *P* = 0.01057). Dietary intake of carbohydrate and fiber were negatively correlated with sUA (β = − 0.001, 95% CI: − 0.002 to − 0.000, *P* < 0.00001; β = − 0.008, 95% CI: − 0.011 to − 0.004, *P* = 0.00001; respectively). Moreover, systolic BP was positively correlated with sUA (β = 0.006, 95% CI: 0.003 to 0.009, *P* = 0.00002). However, no statistical differences were found about the associations of dietary intake of total sugars, protein, total fat, cholesterol, and diastolic BP with sUA.

**Conclusions:**

The current cross-sectional investigation of a nationally representative sample of US participants showed that BMI, dietary energy intake, and systolic BP were positively correlated with sUA levels; dietary carbohydrate and fiber intake were negatively correlated with sUA levels. The findings might be helpful for the management and treatment of hyperuricemia and gout.

**Supplementary Information:**

The online version contains supplementary material available at 10.1186/s12902-022-01112-5.

## Background

Uric acid (UA) is a byproduct of purine metabolism and hyperuricemia (HUA) results from an imbalance in UA synthesis and excretion. HUA is becoming a critical issue affecting individuals all over the world, and the critical precursor to gout which is the most common form of inflammatory arthritis affecting 9.2 million adults (3.9%) in the United States (US) [1]. Apart from gout, HUA is proved to be connected with cardiovascular disorders including obesity and hypertension. Clinical investigations of HUA-related risk factors aid in the early detection, prevention, and management of HUA, gout, and even cardiovascular disorders. Ongoing research is examining the correlation of obesity, dietary patterns, and blood pressure (BP) with serum uric acid (sUA).

Currently, obesity and HUA, along with their related health issues, have developed as serious public health concerns as their growing prevalence. Obesity and HUA coexistence may accelerate disease progression, resulting in a higher medical and economic burden, posing additional difficulties to chronic disease prevention and treatment. The link between sUA and obesity can be explained by several mechanisms including the increased liver synthesis, insulin resistance, endocrine role of adipokines, and genetic factors. The earlier epidemiological and clinical evidence has shown a positive connection between obesity and sUA in Chinese, Japanese, Indian, Pakistani, Iraqi, and Bangladeshi populations [2–4]. However, there is a scarcity of research on the correlation between sUA and obesity based on a nationally representative sample of US participants. Furthermore, sUA levels are greatly influenced by dietary factors. Current guidelines recommend dietary recommendations including limiting purine intake and high-fructose corn syrup intake [5]. However, study on the influence of other dietary factors such as dietary intake of energy, carbohydrate, protein, total fat, cholesterol, and fiber on UA was limited and controversial. Moreover, the close relationship between HUA and hypertension is not a recent observation [6–8]. At present, many studies focus on the effect of UA on hypertension. However, there are few studies investigating the influence of BP on sUA. The National Health and Nutrition Examination Survey (NHANES) is a key initiative of the National Center for Health Data in the US, which is part of the Centers for Disease Control and Prevention and is in charge of producing essential and medical statistical data for the whole nation. To date, based on NHANES data, there is a scarcity of research on the correlation of obesity, dietary patterns, and BP with sUA levels.

Given the probable association of obesity, dietary patterns, and BP with sUA, and their role in the management and treatment of HUA, gout, and cardiovascular diseases, the present study aimed to assess the correlation of obesity, dietary patterns, and BP with sUA based on the 2017–2018 NHANES data.

## Materials and methods

### Study population

The NHANES is a typical survey including the national subjects in the US and provides a plethora of data about the nutrition and health of the general US population by employing a complex and multistage sampling technique. The present study included NHANES data between 2017 and 2018 (*n* = 9254), which represented one cycle. After excluding individuals with missing sUA (*n* = 3353), body mass index (BMI) (*n* = 1249), dietary patterns (dietary intake of energy, carbohydrate, total sugars, protein, total fat, cholesterol, and fiber) (*n* = 1770), and BP (systolic BP and diastolic BP) (*n* = 2952). Finally, 5809, 5393, and 5250 subjects were included in the outcome analysis of the correlation of BMI, dietary patterns, and BP with sUA, respectively.

### Measurements of covariates

The exposure variable was sUA (mg/dL). The Roche Cobas 6000 (c501 module) technique was used to measure sUA between 2017 and 2018. The outcome variable was BMI, dietary patterns, and BP. BMI was calculated using the formula of weight (kg)/height (m)^2^. The data on dietary patterns were obtained from detailed dietary intake information of NHANES participants through dietary interviews. BP data was the average of 3 consecutive BP readings.

Categorical variables of covariates included in our analysis were as follows: gender (male or female), race/ethnicity (Mexican American, other Hispanic, non-Hispanic white, non-Hispanic black, or other race), education levels (less than high school, high school, or more than high school), marital status (living with a spouse or partner: yes or no), alcohol consumption (yes or no), and smoking behavior (yes or no). Continuous covariates: age (years), poverty to income ratio, weight (kg), height (m), waist circumference (cm), hip circumference (cm), minutes sedentary activity (minutes), total cholesterol (mg/dL), triglyceride (mg/dL), high-density lipoprotein cholesterol (mg/dL), low-density lipoprotein cholesterol (mg/dL), fasting blood glucose (mg/dL), and glycohemoglobin (%). The criteria for selecting covariates were based on previously published research and variables [9]. The complete data on sUA, BMI, dietary patterns, BP, and confounders can be found at http://www.cdc.gov/nchs/nhanes/.

### Statistical analysis

To account for the significant volatility in our data set, we utilized a weighted and variance estimation strategy. To assess the correlation of sUA with BMI, dietary patterns, and BP, a weighted multivariate logistic regression model was utilized. To count the discrepancies between subgroups, we applied the weighted χ2 test for the categorical data and the weighted linear regression model for the continuous variables. The stratified multivariate regression analysis was used to accomplish the subgroup analysis. Additionally, smooth curve fittings and generalized additive models were applied to clarify the non-linear relation of sUA with BMI, dietary patterns, and BP. When nonlinearity was identified, the inflection point in the connection of sUA with BMI, dietary patterns, and BP was estimated by a recursive technique, and a two-piecewise linear regression model was performed on both sides of the inflection point. In addition, we conducted the multiplicative interaction analysis and calculated the interaction *P*-value. Statistical analyses were carried out using the R package (http://www.r-project.org) and EmpowerStats (http://www.empowerstats.com), with a *P* < 0.05 threshold deemed statistically significant.

## Results

### Correlation of BMI with sUA

The weighted characteristics subclassified according to sUA quartiles (Q1: 0.8–4.3 mg/dL; Q2: 4.4–5.3 mg/dL; Q3: 5.4–6.3 mg/dL; and Q4: 6.4–15.1 mg/dL) were presented in Table 1. Except for the ratio of family income to poverty, dietary total sugars, dietary fiber, and FBG, there were significant differences in baseline characteristics between the sUA quartiles.

**Table 1:** Weighted characteristics of the study population based on serum uric acid quartiles

Serum uric acid (mg/dL)	ALL (5.40 ± 1.48)	Q1 (0.8–4.3)	Q2 (4.4–5.3)	Q3 (5.4–6.3)	Q4 (6.4–15.1)	***P*** value
Age (years)	45.36 ± 20.93	41.23 ± 18.45	42.47 ± 19.01	46.80 ± 19.36	47.90 ± 19.46	< 0.0001
Gender (%)						< 0.0001
Male	48.49	15.15	38.97	61.43	77.24	
Female	51.51	84.85	61.03	38.57	22.76	
Race/ethnicity (%)						0.0151
Mexican American	14.62	11.72	9.37	9.22	9.15	
Other Hispanic	9.12	7.68	7.23	6.89	6.25	
Non-Hispanic White	34.41	60.39	61.92	64.54	60.03	
Non-Hispanic Black	22.28	11.11	10.66	9.23	12.49	
Other races	19.57	9.09	10.81	10.12	12.07	
Alcohol consumption (%)						0.0004
< 12 drinks daily	55.1	61.77	66.20	64.50	67.96	
> = 12 drinks daily	0.96	0.65	0.78	1.07	1.71	
Unknown	43.93	37.58	33.02	34.43	30.33	
Smoking behavior (%)						< 0.0001
Smoked at least 100 cigarettes in life	35.36	31.27	36.84	41.41	41.77	
Smoked less than 100 cigarettes in life	51.89	56.86	53.67	50.57	53.20	
Unknown	12.76	11.87	9.49	8.02	5.04	
Education level (%)						< 0.0001
Less than high school	16.39	9.98	9.57	9.35	10.73	
High school	19.78	20.07	23.77	27.02	25.04	
More than high school	46.94	55.17	53.80	53.03	56.35	
Unknown	16.89	14.78	12.86	10.60	7.89	
Marital status (%)						< 0.0001
Live with a partner	49.32	56.78	52.46	55.98	57.93	
Live alone	33.86	28.44	34.82	33.50	34.16	
Unknown	16.82	14.78	12.72	10.52	7.91	
Ratio of family income to poverty	2.49 ± 1.50	2.92 ± 1.56	2.96 ± 1.57	2.99 ± 1.58	3.00 ± 1.58	0.5494
Energy (kcal/day)	2104.84 ± 989.21	2030.98 ± 852.99	2132.82 ± 932.57	2218.70 ± 1025.26	2311.58 ± 1079.23	< 0.0001
Dietary carbohydrate (g/day)	251.482 ± 124.763	241.356 ± 112.176	247.346 ± 119.203	257.552 ± 130.022	259.538 ± 135.375	0.00017
Dietary total sugars (g/day)	109.204 ± 76.448	106.527 ± 69.271	107.022 ± 68.857	111.079 ± 82.457	112.281 ± 84.127	0.10547
Dietary protein (g/day)	81.865 ± 41.721	76.144 ± 38.570	79.999 ± 40.328	83.847 ± 42.230	87.346 ± 44.668	< 0.0001
Dietary total fat (g/day)	88.808 ± 48.124	83.070 ± 42.867	87.819 ± 45.869	89.997 ± 48.489	94.125 ± 53.912	< 0.0001
Dietary cholesterol (mg/day)	305.893 ± 234.990	275.258 ± 210.860	304.657 ± 239.832	301.944 ± 221.416	340.119 ± 258.753	< 0.0001
Dietary fiber (g/day)	16.477 ± 10.053	16.010 ± 9.437	16.642 ± 10.569	16.941 ± 10.225	16.264 ± 9.822	0.07097
Minutes sedentary activity (minutes)	377.44 ± 659.37	385.09 ± 658.06	379.14 ± 550.71	444.36 ± 971.51	376.10 ± 330.59	0.0168
Body mass index (kg/m^2^)	29.04 ± 7.53	26.30 ± 6.26	28.47 ± 7.32	30.02 ± 7.08	31.97 ± 7.52	< 0.0001
Glycohemoglobin (%)	5.77 ± 1.04	5.59 ± 1.07	5.60 ± 0.80	5.63 ± 0.80	5.72 ± 0.88	0.0003
Fasting blood glucose (mg/dL)	111.95 ± 24.57	109.85 ± 23.51	109.81 ± 22.26	110.80 ± 18.83	111.36 ± 20.10	0.1372
Total cholesterol (mg/dL)	182.92 ± 41.30	182.27 ± 39.59	184.71 ± 40.85	184.79 ± 40.03	189.83 ± 42.25	< 0.0001
Triglyceride (mg/dL)	107.78 ± 68.47	97.85 ± 38.49	104.11 ± 49.36	108.04 ± 58.99	121.90 ± 96.51	< 0.0001
High-density lipoprotein cholesterol (mg/dL)	53.04 ± 15.06	58.72 ± 14.56	55.31 ± 15.13	50.89 ± 14.10	48.81 ± 14.11	< 0.0001
Low-density lipoprotein cholesterol (mg/dL)	106.95 ± 24.47	106.05 ± 22.34	107.72 ± 23.53	107.66 ± 24.95	109.14 ± 25.61	0.0098
Systolic blood pressure (mm Hg)	123.41 ± 18.22	117.80 ± 17.07	120.00 ± 16.00	123.52 ± 15.84	124.90 ± 16.25	< 0.0001
Diastolic blood pressure (mm Hg)	70.18 ± 13.17	69.27 ± 11.58	69.82 ± 13.06	71.40 ± 12.14	72.57 ± 12.08	< 0.0001

Mean ± SD for continuous variables: the *P* value was calculated by the weighted linear regression model. (%) for categorical variables: the *P* value was calculated by the weighted chi-square test

Table 2 presented the findings of the multivariate regression analysis. BMI was positively correlated with sUA in the unadjusted model 1 (β = 0.057, 95% CI: 0.053 to 0.062, *P* < 0.00001). After adjusting for covariates, this significant link remained in model 2 (β = 0.058, 95% CI: 0.053 to 0.062, *P* < 0.00001) and 3 (β = 0.059, 95% CI: 0.054 to 0.064, *P* < 0.00001). Furthermore, overweight and obese individuals had higher sUA levels than those with normal BMI (β = 0.451, 95% CI: 0.357 to 0.546, *P* < 0.00001; β = 0.853, 95% CI: 0.760 to 0.946, *P* < 0.00001; respectively). According to the results of subgroup analyses stratified by gender and race, the positive association of BMI with sUA remained in both males (β = 0.061, 95% CI: 0.052 to 0.070, *P* < 0.00001) and females (β = 0.057, 95% CI: 0.051 to 0.063, *P* < 0.00001), as well as in all races including Mexican American (β = 0.051, 95% CI: 0.036 to 0.066, *P* < 0.00001), other Hispanic (β = 0.055, 95% CI: 0.034 to 0.075, *P* < 0.00001), non-Hispanic White (β = 0.061, 95% CI: 0.053 to 0.070, *P* < 0.00001), non-Hispanic Black (β = 0.061, 95% CI: 0.051 to 0.072, *P* < 0.00001), and other races (β = 0.046, 95% CI: 0.033 to 0.059, *P* < 0.00001).

**Table 2:** The association between BMI (kg/m^2^) and serum uric acid (mg/dL)

	Model 1 β (95% CI) ***P*** value	Model 2 β (95% CI) ***P*** value	Model 3 β (95% CI) ***P*** value
BMI (kg/m^2^)	0.057 (0.053, 0.062) < 0.00001	0.058 (0.053, 0.062) < 0.00001	0.059 (0.054, 0.064) < 0.00001
BMI categories			
Normal BMI (< 25 kg/m^2^)	Reference	Reference	Reference
Overweight (25 to 30 kg/m^2^)	0.676 (0.586, 0.767) < 0.00001	0.484 (0.402, 0.566) < 0.00001	0.451 (0.357, 0.546) < 0.00001
Obesity (> = 30 kg/m^2^)	1.004 (0.920, 1.089) < 0.00001	0.896 (0.820, 0.973) < 0.00001	0.853 (0.760, 0.946) < 0.00001
Subgroup analysis stratified by gender			
Male	0.063 (0.056, 0.070) < 0.00001	0.063 (0.056, 0.070) < 0.00001	0.061 (0.052, 0.070) < 0.00001
Female	0.058 (0.053, 0.063) < 0.00001	0.055 (0.050, 0.060) < 0.00001	0.057 (0.051, 0.063) < 0.00001
Subgroup analysis stratified by race/ethnicity			
Mexican American	0.053 (0.039, 0.067) < 0.00001	0.057 (0.045, 0.069) < 0.00001	0.051 (0.036, 0.066) < 0.00001
Other Hispanic	0.064 (0.045, 0.083) < 0.00001	0.056 (0.040, 0.072) < 0.00001	0.055 (0.034, 0.075) < 0.00001
Non-Hispanic White	0.060 (0.052, 0.068) < 0.00001	0.057 (0.050, 0.064) < 0.00001	0.061 (0.053, 0.070) < 0.00001
Non-Hispanic Black	0.052 (0.042, 0.061) < 0.00001	0.062 (0.053, 0.070) < 0.00001	0.061 (0.051, 0.072) < 0.00001
Other races	0.063 (0.052, 0.074) < 0.00001	0.058 (0.048, 0.068) < 0.00001	0.046 (0.033, 0.059) < 0.00001

Model 1: no covariates were adjusted. Model 2: age, gender, and race/ethnicity were adjusted. Model 3: age, gender, race/ethnicity, alcohol consumption, smoking behavior, education level, marital status, the ratio of family income to poverty, energy, minutes of sedentary activity, glycohemoglobin, fasting glucose, total cholesterol, triglyceride, high-density lipoprotein cholesterol, low-density lipoprotein cholesterol, systolic blood pressure, and diastolic blood pressure were adjusted. In the subgroup analysis stratified by gender and race/ethnicity; the model is not adjusted for gender and race/ethnicity, respectively

**Fig. 1:**
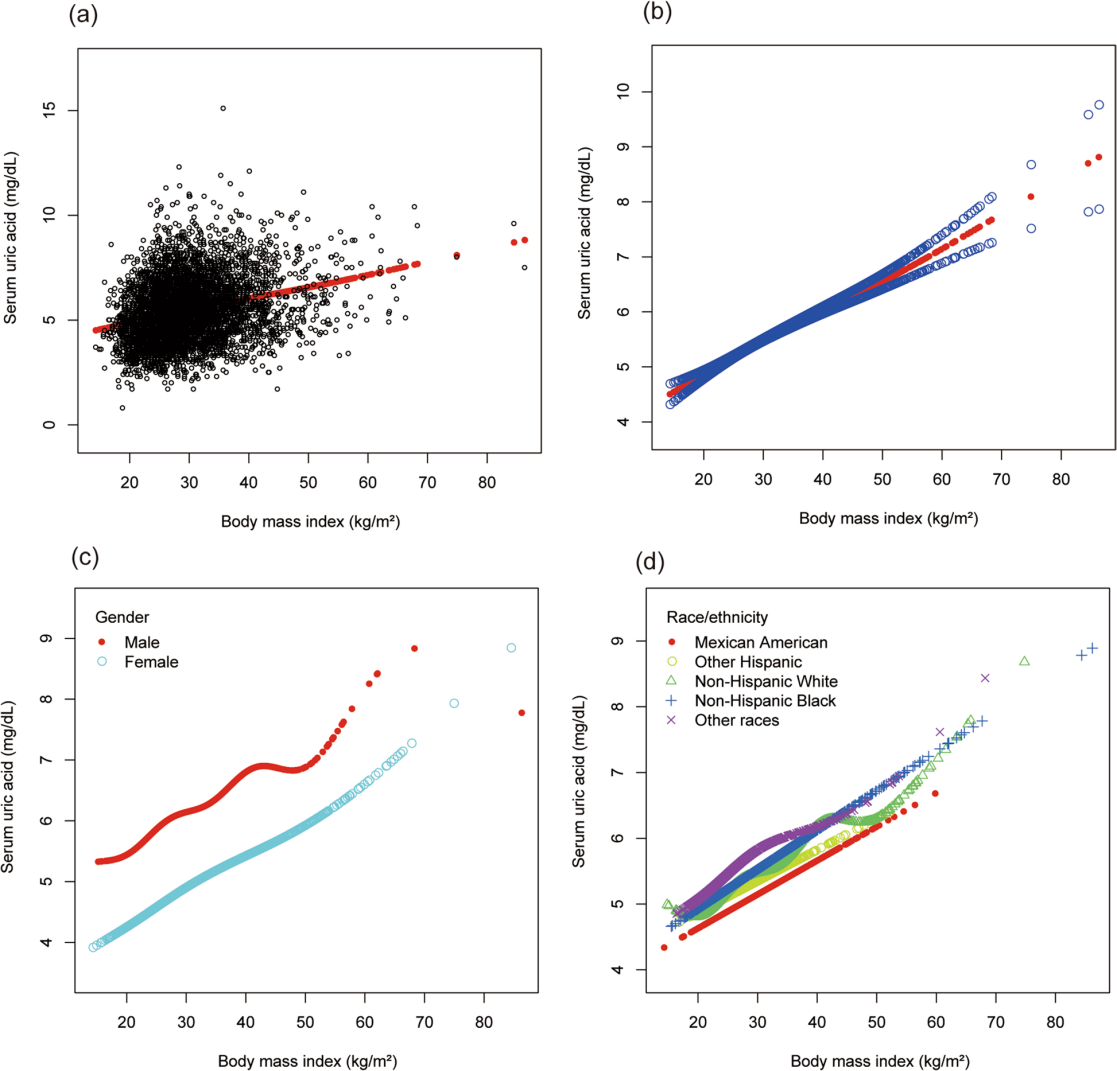
The association between body mass index and serum uric acid. **a** Each black point represents a sample. **b** The solid red line represents the smooth curve fit between variables. **c** Stratified by gender. **d** Stratified by race/ethnicity

To evaluate the nonlinear connection between BMI and sUA stratified by gender and race/ethnicity, smooth curve fittings and generalized additive models were applied (Fig. 1).

### Correlation of dietary patterns with sUA

#### Correlation of dietary energy intake with sUA

As shown in Table 3, dietary energy intake was positively correlated with sUA (β = 0.000, 95% CI: 0.000 to 0.000, *P* = 0.01057). Individuals with higher dietary energy intake (2619 to 12,501 kcal/day) had higher sUA levels than those with lower dietary energy intake (3 to 1401 kcal/day) (β = 0.150, 95% CI: 0.036 to 0.265, *P* = 0.01025). According to the results of subgroup analyses stratified by gender and race, the positive association of dietary energy intake with sUA remained in the subgroup of other races (β = 0.000, 95% CI: 0.000 to 0.000, *P* = 0.01609).

**Table 3:** The association between dietary energy intake (kcal/day) and serum uric acid (mg/dL)

	Model 1 β (95% CI) ***P*** value	Model 2 β (95% CI) ***P*** value	Model 3 β (95% CI) ***P*** value
Dietary energy intake (kcal/day)	0.000 (0.000, 0.000) < 0.00001	0.000 (0.000, 0.000) < 0.00001	0.000 (0.000, 0.000) 0.01057
Dietary energy intake categories			
Q1 (3 to 1401 kcal/day)	Reference	Reference	Reference
Q2 (1402 to 1933 kcal/day)	−0.029 (− 0.141, 0.082) 0.60497	− 0.029 (− 0.139, 0.081) 0.60775	−0.062 (− 0.177, 0.053) 0.29249
Q3 (1934 to 2618 kcal/day)	0.091 (− 0.018, 0.200) 0.10076	0.108 (0.000, 0.216) 0.04964	− 0.007 (− 0.121, 0.107) 0.90356
Q4 (2619 to 12,501 kcal/day)	0.369 (0.261, 0.477) < 0.00001	0.402 (0.295, 0.510) < 0.00001	0.150 (0.036, 0.265) 0.01025
Subgroup analysis stratified by gender			
Male	−0.000 (− 0.000, − 0.000) 0.03811	−0.000 (− 0.000, 0.000) 0.05576	−0.000 (− 0.000, 0.000) 0.05735
Female	− 0.000 (− 0.000, − 0.000) 0.00078	−0.000 (− 0.000, − 0.000) 0.01701	−0.000 (− 0.000, 0.000) 0.06202
Subgroup analysis stratified by race/ethnicity			
Mexican American	0.000 (0.000, 0.000) 0.00741	0.000 (0.000, 0.000) 0.00594	0.000 (−0.000, 0.000) 0.59359
Other Hispanic	0.000 (0.000, 0.000) 0.00351	0.000 (0.000, 0.000) 0.00066	0.000 (−0.000, 0.000) 0.42501
Non-Hispanic White	0.000 (0.000, 0.000) < 0.00001	0.000 (0.000, 0.000) < 0.00001	0.000 (−0.000, 0.000) 0.15089
Non-Hispanic Black	−0.000 (− 0.000, 0.000) 0.71615	0.000 (− 0.000, 0.000) 0.41964	0.000 (− 0.000, 0.000) 0.50106
Other races	0.000 (0.000, 0.000) < 0.00001	0.000 (0.000, 0.000) < 0.00001	0.000 (0.000, 0.000) 0.01609

Model 1: no covariates were adjusted. Model 2: age, gender, and race/ethnicity were adjusted. Model 3: age, gender, race/ethnicity, alcohol consumption, smoking behavior, education level, marital status, the ratio of family income to poverty, minutes of sedentary activity, body mass index, waist circumference, hip circumference, glycohemoglobin, fasting glucose, total cholesterol, triglyceride, high-density lipoprotein cholesterol, low-density lipoprotein cholesterol, systolic blood pressure, and diastolic blood pressure were adjusted. In the subgroup analysis stratified by gender and race/ethnicity; the model is not adjusted for gender and race/ethnicity, respectively

**Fig. 2:**
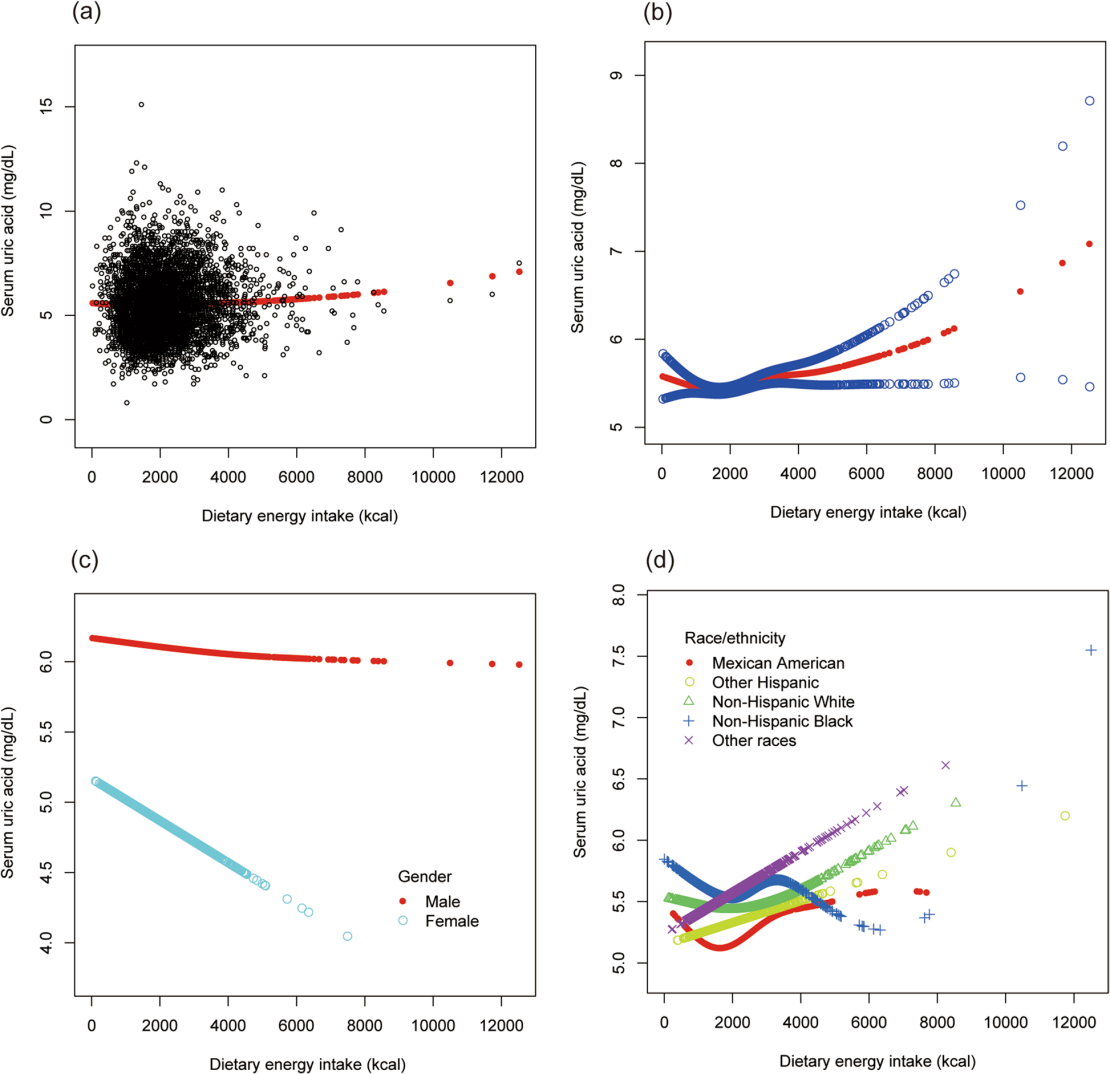
The association between dietary energy intake and serum uric acid. **a** Each black point represents a sample. **b** The solid red line represents the smooth curve fit between variables. Blue bands represent the 95% of confidence interval from the fit. **c** Stratified by gender. **d** Stratified by race/ethnicity

To evaluate the nonlinear connection between dietary energy intake and sUA stratified by gender and race/ethnicity, smooth curve fittings and generalized additive models were applied (Fig. 2).

#### Correlation of dietary carbohydrate with sUA

Dietary carbohydrate intake was positively correlated with sUA in the unadjusted model (β = 0.001, 95% CI: 0.000 to 0.001, *P* = 0.00009); after adjusting for covariates, dietary carbohydrate intake was negatively correlated with sUA in model 2 (β = − 0.001, 95% CI: − 0.001 to − 0.000, *P* < 0.00001) and 3 (β = − 0.001, 95% CI: − 0.002 to − 0.000, *P* < 0.00001) (Table 4). Furthermore, individuals with higher dietary carbohydrate intake had lower sUA levels than those with lower dietary carbohydrate intake. According to the results of subgroup analyses stratified by gender and race, the negative association of dietary carbohydrate intake with sUA remained in men (β = − 0.001, 95% CI: − 0.002, − 0.000, *P* = 0.00393), as well as in races of non-Hispanic White (β = − 0.001, 95% CI: − 0.002 to − 0.000, *P* = 0.00389) and other races (β = − 0.001, 95% CI: − 0.003 to − 0.000, *P* = 0.01253) (Table 4).

**Table 4:** The association between dietary carbohydrate (g/day) and serum uric acid (mg/dL)

	Model 1 β (95% CI) ***P*** value	Model 2 β (95% CI) ***P*** value	Model 3 β (95% CI) ***P*** value
Dietary carbohydrate (g/day)	0.001 (0.000, 0.001) 0.00009	−0.001 (− 0.001, − 0.000) < 0.00001	− 0.001 (− 0.002, − 0.000) 0.00040
Dietary carbohydrate categories			
Q1 (1.1–161.16 g/day)	Reference	Reference	Reference
Q2 (161.17–226.13 g/day)	− 0.112 (− 0.221, − 0.003) 0.04335	−0.215 (− 0.311, − 0.119) 0.00001	−0.175 (− 0.279, − 0.071) 0.00096
Q3 (226.14–312.56 g/day)	− 0.072 (− 0.179, 0.035) 0.18429	−0.278 (− 0.373, − 0.183) < 0.00001	−0.273 (− 0.388, − 0.159) < 0.00001
Q4 (312.57–1476.76 g/day)	0.168 (0.061, 0.276) 0.00211	−0.269 (− 0.367, − 0.171) < 0.00001	− 0.296 (− 0.446, − 0.146) 0.00011
Subgroup analysis stratified by gender			
Male	− 0.001 (− 0.001, − 0.000) 0.00001	− 0.001 (− 0.001, − 0.000) 0.00002	−0.001 (− 0.002, − 0.000) 0.00393
Female	−0.001 (− 0.001, − 0.000) 0.00019	−0.001 (− 0.001, − 0.000) 0.00592	−0.001 (− 0.002, 0.000) 0.11313
Subgroup analysis stratified by race/ethnicity			
Mexican American	0.001 (0.000, 0.002) 0.01029	−0.000 (− 0.001, 0.000) 0.28091	0.002 (− 0.000, 0.004) 0.05702
Other Hispanic	0.001 (0.001, 0.002) 0.00110	−0.000 (− 0.001, 0.001) 0.80984	0.000 (− 0.002, 0.002) 0.75684
Non-Hispanic White	0.001 (0.000, 0.001) 0.01795	− 0.001 (− 0.001, − 0.000) 0.00119	−0.001 (− 0.002, − 0.000) 0.00389
Non-Hispanic Black	−0.000 (− 0.001, 0.000) 0.21029	−0.001 (− 0.002, − 0.000) 0.00153	−0.001 (− 0.002, 0.000) 0.14506
Other races	0.001 (0.000, 0.001) 0.03012	−0.001 (− 0.001, − 0.000) 0.02849	−0.001 (− 0.003, − 0.000) 0.01253

Model 1: no covariates were adjusted. Model 2: age, gender, and race/ethnicity were adjusted. Model 3: age, gender, race/ethnicity, alcohol consumption, smoking behavior, education level, marital status, the ratio of family income to poverty, energy, minutes of sedentary activity, weight, height, body mass index, waist circumference, hip circumference, glycohemoglobin, fasting glucose, total cholesterol, triglyceride, high-density lipoprotein cholesterol, low-density lipoprotein cholesterol, systolic blood pressure, and diastolic blood pressure were adjusted. In the subgroup analysis stratified by gender and race/ethnicity; the model is not adjusted for gender and race/ethnicity, respectively

**Fig. 3:**
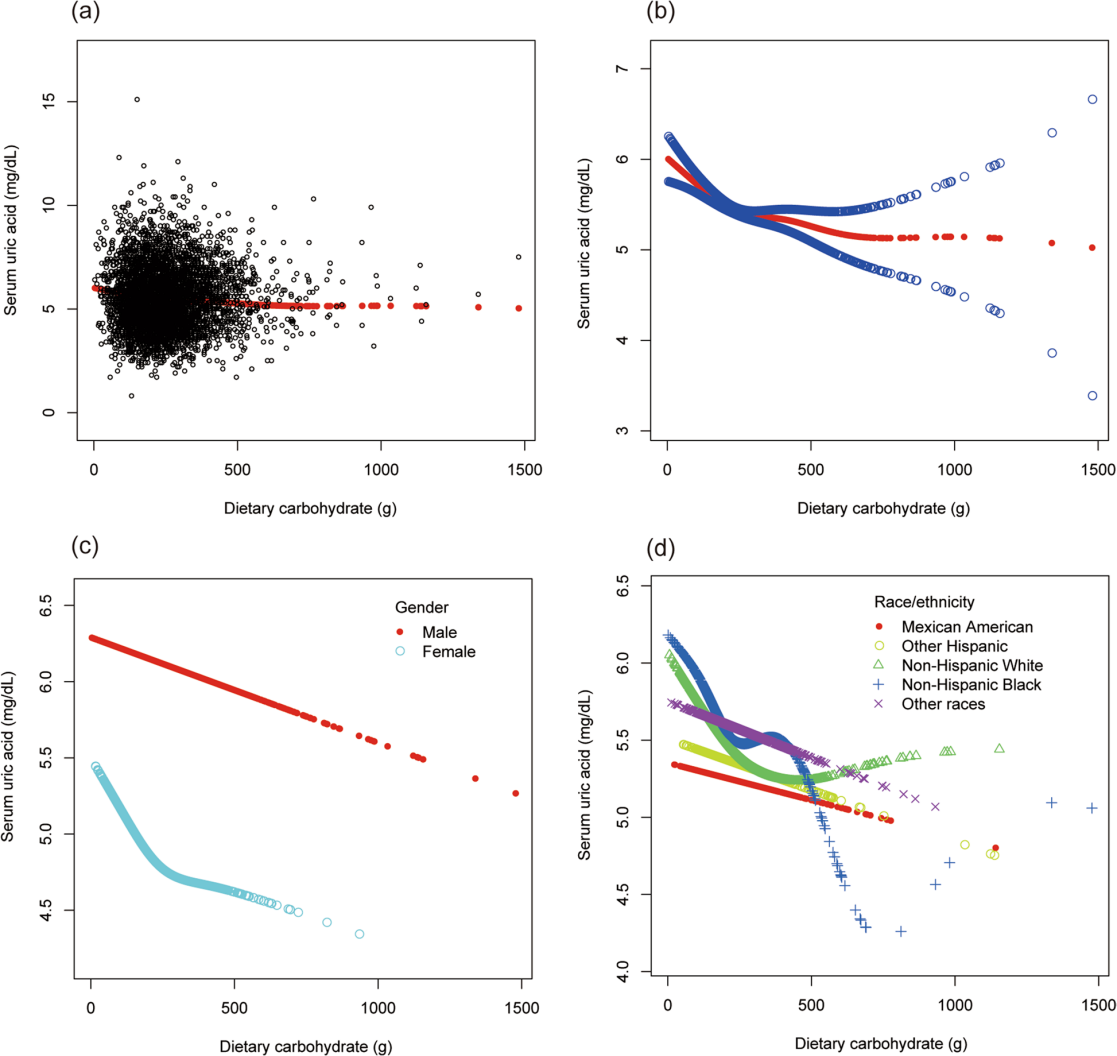
The association between dietary carbohydrate and serum uric acid. **a** Each black point represents a sample. **b** The solid red line represents the smooth curve fit between variables. Blue bands represent the 95% of confidence interval from the fit. **c** Stratified by gender. **d** Stratified by race/ethnicity

To evaluate the nonlinear connection between dietary carbohydrate and sUA stratified by gender and race/ethnicity, smooth curve fittings and generalized additive models were applied (Fig. 3).

#### Correlation of dietary total sugars with sUA

As presented in Table 5, no statistical difference was found about the associations of dietary total sugars intake with sUA (β = − 0.001, 95% CI: − 0.001 to 0.000, *P* = 0.06756). However, individuals with higher dietary sugars intake (90.89 to 138.19 g/day or 138.20 to 931.16 g/day) had lower sUA levels (β = − 0.126, 95% CI: − 0.230 to − 0.023, *P* = 0.01655; β = − 0.192, 95% CI: − 0.311 to − 0.073, *P* = 0.00160; respectively). According to the results of subgroup analyses stratified by gender and race, no statistical difference in the associations of dietary sugar intake with sUA was found in different gender and races. According to the results of interaction analysis, the relationship between dietary total sugar intake and sUA was significantly different in the non-Hispanic White subgroup (P interaction < 0.05) (Supplementary Table 1).

**Table 5:** The association between dietary sugars (g/day) and serum uric acid (mg/dL)

	Model 1 β (95% CI) ***P*** value	Model 2 β (95% CI) ***P*** value	Model 3 β (95% CI) ***P*** value
Dietary sugars (g/day)	0.000 (− 0.000, 0.001) 0.08544	− 0.001 (− 0.001, − 0.000) 0.00049	− 0.001 (− 0.001, 0.000) 0.06756
Dietary sugars categories			
Q1 (0 to 56.03 g/day)	Reference	Reference	Reference
Q2 (56.04 to 90.88 g/day)	−0.102 (− 0.211, 0.007) 0.06632	−0.144 (− 0.240, − 0.048) 0.00324	−0.092 (− 0.193, 0.009) 0.07381
Q3 (90.89 to 138.19 g/day)	− 0.024 (− 0.131, 0.084) 0.66537	−0.176 (− 0.271, − 0.082) 0.00026	−0.126 (− 0.230, − 0.023) 0.01655
Q4 (138.20 to 931.16 g/day)	0.017 (− 0.090, 0.124) 0.75448	− 0.239 (− 0.334, − 0.144) < 0.00001	− 0.192 (− 0.311, − 0.073) 0.00160
Subgroup analysis stratified by gender			
Male	− 0.001 (− 0.001, − 0.000) 0.00694	− 0.001 (− 0.001, − 0.000) 0.00885	− 0.000 (− 0.001, 0.000) 0.37218
Female	− 0.001 (− 0.002, − 0.000) 0.00725	−0.001 (− 0.001, − 0.000) 0.02971	−0.000 (− 0.001, 0.000) 0.34858
Subgroup analysis stratified by race/ethnicity			
Mexican American	0.003 (0.001, 0.004) 0.00021	0.001 (−0.000, 0.003) 0.05444	0.003 (−0.002, 0.005) 0.05018
Other Hispanic	0.002 (0.000, 0.003) 0.02712	0.000 (−0.001, 0.002) 0.76162	0.001 (−0.001, 0.003) 0.39065
Non-Hispanic White	0.000 (−0.001, 0.001) 0.75266	−0.001 (− 0.002, − 0.000) 0.00401	−0.001 (− 0.002, 0.000) 0.05291
Non-Hispanic Black	− 0.000 (− 0.002, 0.001) 0.38480	−0.001 (− 0.002, − 0.000) 0.02064	−0.001 (− 0.003, 0.000) 0.12336
Other races	0.001 (− 0.000, 0.002) 0.12889	−0.001 (− 0.002, 0.001) 0.33848	−0.001 (− 0.002, 0.000) 0.18799

Model 1: no covariates were adjusted. Model 2: age, gender, and race/ethnicity were adjusted. Model 3: age, gender, race/ethnicity, alcohol consumption, smoking behavior, education level, marital status, the ratio of family income to poverty, energy, minutes of sedentary activity, weight, height, body mass index, waist circumference, hip circumference, glycohemoglobin, fasting glucose, total cholesterol, triglyceride, high-density lipoprotein cholesterol, low-density lipoprotein cholesterol, systolic blood pressure, and diastolic blood pressure were adjusted. In the subgroup analysis stratified by gender and race/ethnicity; the model is not adjusted for gender and race/ethnicity, respectively

**Fig. 4:**
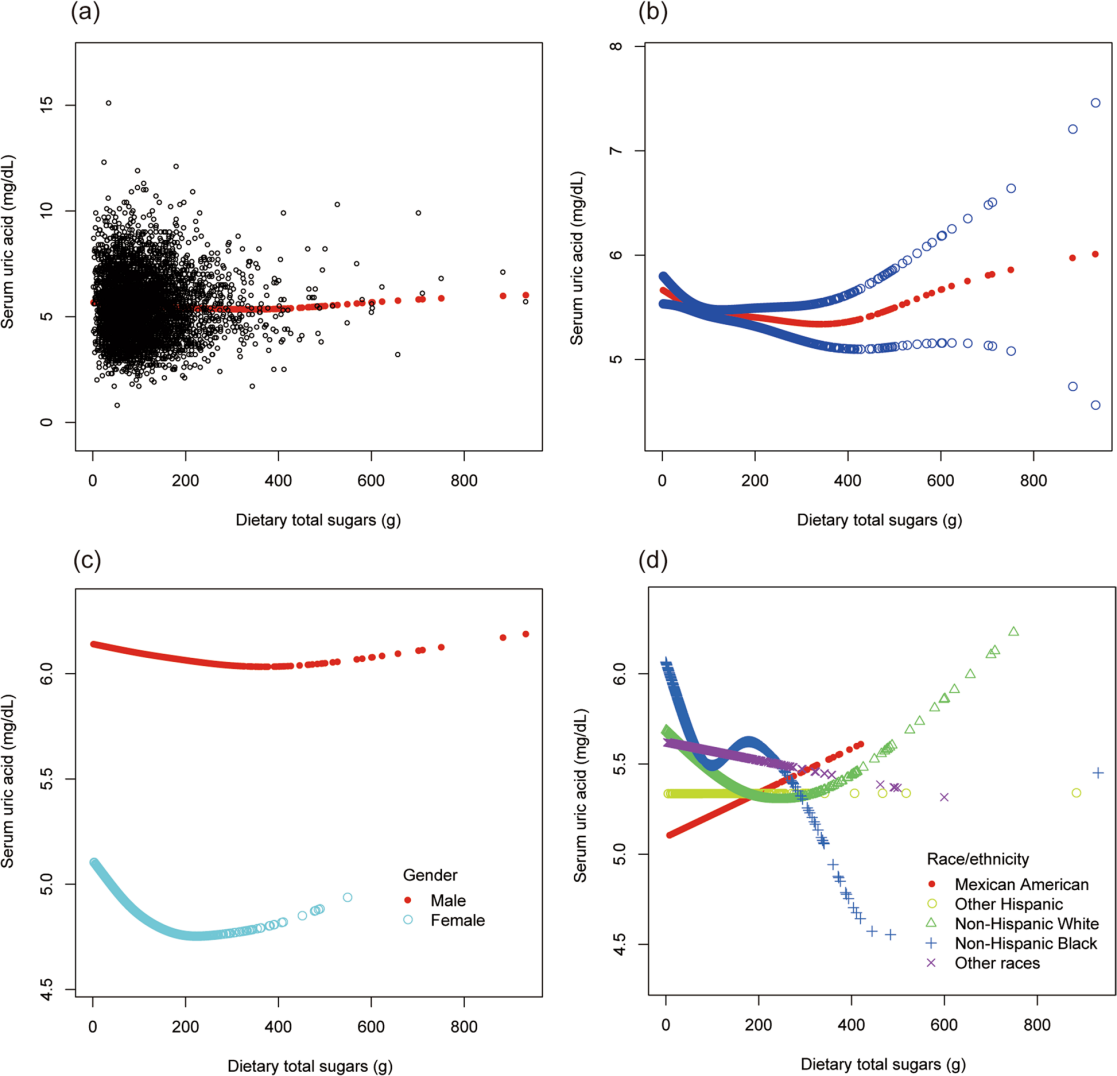
The association between dietary total sugars and serum uric acid. **a** Each black point represents a sample. **b** The solid red line represents the smooth curve fit between variables. Blue bands represent the 95% of confidence interval from the fit. **c** Stratified by gender. **d** Stratified by race/ethnicity

To evaluate the nonlinear connection between dietary sugar intake and sUA stratified by gender and race/ethnicity, smooth curve fittings and generalized additive models were applied (Fig. 4).

#### Correlation of dietary protein with sUA

No statistical difference was found about the associations of dietary protein intake with sUA (β = − 0.001, 95% CI: − 0.002 to 0.000, *P* = 0.12523) (Table 6). Furthermore, no statistical difference about the associations of dietary protein intake with sUA was found in different dietary protein categories. However, dietary protein intake was negatively correlated with sUA in male (β = − 0.002, 95% CI: − 0.004 to − 0.000, *P* = 0.01696), Mexican American (β = − 0.004, 95% CI: − 0.008 to − 0.001, *P* = 0.01599), and other Hispanic (β = − 0.005, 95% CI: − 0.009 to − 0.000, *P* = 0.03886) (Table 6).

**Table 6:** The association between dietary protein (g) and serum uric acid (mg/dL)

	Model 1 β (95% CI) ***P*** value	Model 2 β (95% CI) ***P*** value	Model 3 β (95% CI) ***P*** value
Dietary protein (g)	0.004 (0.003, 0.004) < 0.00001	− 0.001 (− 0.002, − 0.000) 0.00307	−0.001 (− 0.002, 0.000) 0.12523
Dietary protein categories			
Q1 (0.01–49.79 g)	Reference	Reference	Reference
Q2 (49.80–70.88 g)	−0.028 (− 0.139, 0.083) 0.62399	−0.106 (− 0.205, − 0.007) 0.03543	−0.070 (− 0.178, 0.037) 0.20139
Q3 (70.89–98.57 g)	0.264 (0.155, 0.373) < 0.00001	0.013 (− 0.085, 0.110) 0.79922	0.069 (− 0.045, 0.183) 0.23456
Q4 (98.58–545.20 g)	0.448 (0.341, 0.554) < 0.00001	− 0.084 (− 0.183, 0.016) 0.09949	−0.011 (− 0.150, 0.127) 0.87306
Subgroup analysis stratified by gender			
Male	−0.001 (− 0.002, − 0.000) 0.01170	−0.001 (− 0.002, − 0.000) 0.01478	−0.002 (− 0.004, − 0.000) 0.01696
Female	−0.002 (− 0.003, − 0.000) 0.02719	−0.001 (− 0.003, 0.000) 0.05537	0.000 (− 0.002, 0.002) 0.73194
Subgroup analysis stratified by race/ethnicity			
Mexican American	0.002 (−0.000, 0.004) 0.12294	− 0.003 (− 0.005, − 0.001) 0.00479	−0.004 (− 0.008, − 0.001) 0.01599
Other Hispanic	0.003 (0.001, 0.006) 0.00754	−0.001 (− 0.004, 0.001) 0.19567	−0.005 (− 0.009, − 0.000) 0.03886
Non-Hispanic White	0.004 (0.003, 0.006) < 0.00001	−0.001 (− 0.003, 0.000) 0.12136	−0.001 (− 0.003, 0.001) 0.31388
Non-Hispanic Black	0.001 (− 0.001, 0.003) 0.45050	−0.002 (− 0.004, 0.000) 0.06558	0.001 (− 0.002, 0.005) 0.43506
Other races	0.006 (0.004, 0.008) < 0.00001	0.001 (− 0.001, 0.003) 0.43113	0.001 (− 0.002, 0.004) 0.44124

Model 1: no covariates were adjusted. Model 2: age, gender, and race/ethnicity were adjusted. Model 3: age, gender, race/ethnicity, alcohol consumption, smoking behavior, education level, marital status, the ratio of family income to poverty, energy, minutes of sedentary activity, weight, height, body mass index, waist circumference, hip circumference, glycohemoglobin, fasting glucose, total cholesterol, triglyceride, high-density lipoprotein cholesterol, low-density lipoprotein cholesterol, systolic blood pressure, and diastolic blood pressure were adjusted. In the subgroup analysis stratified by gender and race/ethnicity; the model is not adjusted for gender and race/ethnicity, respectively

**Fig. 5:**
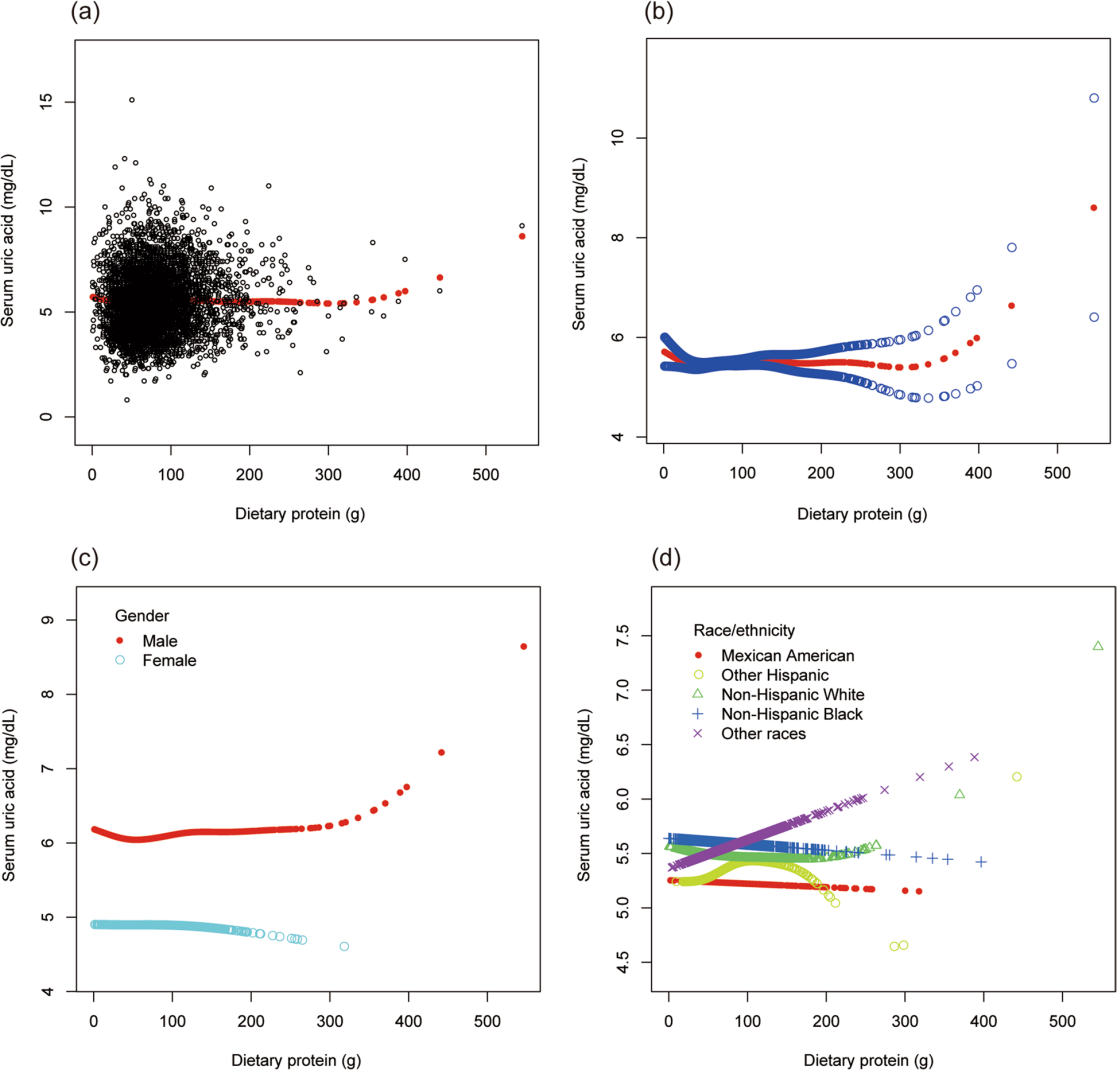
The association between dietary protein and serum uric acid. **a** Each black point represents a sample. **b** The solid red line represents the smooth curve fit between variables. Blue bands represent the 95% of confidence interval from the fit. **c** Stratified by gender. **d** Stratified by race/ethnicity

To evaluate the nonlinear connection between dietary protein intake and sUA stratified by gender and race/ethnicity, smooth curve fittings and generalized additive models were applied (Fig. 5).

#### Correlation of dietary total fat intake with sUA

As shown in Table 7, no statistical difference was found about the associations of dietary total fat intake with sUA (β = 0.000, 95% CI: − 0.001 to 0.001, *P* = 0.61773). However, individuals with highest dietary total fat intake (108.11 to 576.96 g/day) had higher sUA levels than those with lower dietary total fat intake (<= 50.74 g/day) (β = 0.146, 95% CI: 0.028 to 0.264, *P* = 0.01500). Furthermore, no statistical difference was found in different gender and races.

**Table 7:** The association between dietary total fat (g/day) and serum uric acid (mg/dL)

	Model 1 β (95% CI) ***P*** value	Model 2 β (95% CI) ***P*** value	Model 3 β (95% CI) ***P*** value
Dietary total fat (g/day)	0.002 (0.002, 0.003) < 0.00001	0.003 (0.002, 0.003) < 0.00001	0.000 (− 0.001, 0.001) 0.61773
Dietary total fat categories			
Q1 (0 to 50.74 g/day)	Reference	Reference	Reference
Q2 (50.75 to 76.01 g/day)	− 0.039 (− 0.151, 0.074) 0.49947	− 0.037 (− 0.148, 0.075) 0.51592	− 0.010 (− 0.129, 0.109) 0.87031
Q3 (76.02 to 108.10 g/day)	0.034 (− 0.077, 0.145) 0.54801	0.051 (− 0.060, 0.161) 0.36716	− 0.026 (− 0.145, 0.092) 0.66302
Q4 (108.11 to 576.96 g/day)	0.242 (0.133, 0.352) 0.00001	0.268 (0.160, 0.377) < 0.00001	0.146 (0.028, 0.264) 0.01500
Subgroup analysis stratified by gender			
Male	−0.001 (− 0.002, 0.000) 0.07933	−0.001 (− 0.002, 0.000) 0.09457	−0.001 (− 0.002, 0.000) 0.07601
Female	− 0.002 (− 0.003, − 0.001) 0.00435	−0.001 (− 0.002, − 0.000) 0.03226	−0.002 (− 0.003, 0.000) 0.07137
Subgroup analysis stratified by race/ethnicity			
Mexican American	0.001 (−0.001, 0.003) 0.23028	0.001 (−0.001, 0.004) 0.19812	− 0.001 (− 0.003, 0.002) 0.55566
Other Hispanic	0.001 (− 0.001, 0.004) 0.21727	0.002 (− 0.000, 0.004) 0.07468	−0.001 (− 0.003, 0.002) 0.59053
Non-Hispanic White	0.003 (0.002, 0.005) < 0.00001	0.003 (0.002, 0.005) < 0.00001	0.000 (− 0.001, 0.002) 0.70952
Non-Hispanic Black	−0.001 (− 0.003, 0.000) 0.10225	−0.000 (− 0.002, 0.001) 0.56746	−0.001 (− 0.002, 0.001) 0.26927
Other races	0.003 (0.001, 0.005) 0.00027	0.003 (0.002, 0.005) 0.00009	0.001 (−0.001, 0.003) 0.19878

Model 1: no covariates were adjusted. Model 2: age, gender, and race/ethnicity were adjusted. Model 3: age, gender, race/ethnicity, alcohol consumption, smoking behavior, education level, marital status, the ratio of family income to poverty, minutes of sedentary activity, waist circumference, hip circumference, glycohemoglobin, fasting glucose, total cholesterol, triglyceride, high-density lipoprotein cholesterol, low-density lipoprotein cholesterol, systolic blood pressure, and diastolic blood pressure were adjusted. In the subgroup analysis stratified by gender and race/ethnicity; the model is not adjusted for gender and race/ethnicity, respectively

**Fig. 6:**
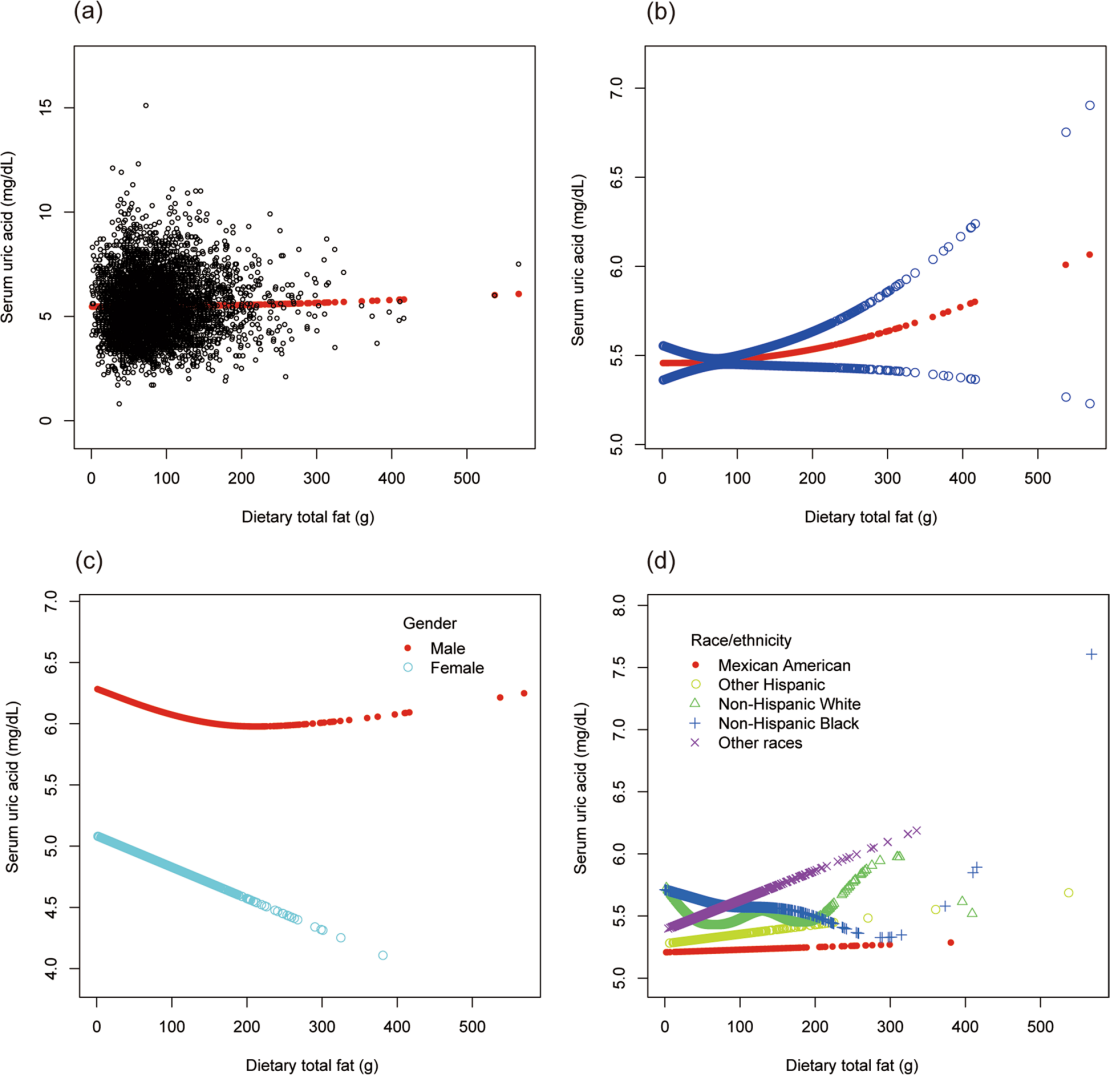
The association between dietary total fat and serum uric acid. **a** Each black point represents a sample. **b** The solid red line represents the smooth curve fit between variables. Blue bands represent the 95% of confidence interval from the fit. **c** Stratified by gender. **d** Stratified by race/ethnicity

To evaluate the nonlinear connection between dietary total fat intake and sUA stratified by gender and race/ethnicity, smooth curve fittings and generalized additive models were applied (Fig. 6).

#### Correlation of dietary cholesterol intake with sUA

No statistical difference was found in the associations of dietary cholesterol intake with sUA (β = − 0.000, 95% CI: − 0.000 to 0.000, *P* = 0.74345) (Table 8). Furthermore, no statistical difference was found in the subgroups of gender and race.

**Table 8:** The association between dietary cholesterol (mg/day) and serum uric acid (mg/dL)

	Model 1 β (95% CI) ***P*** value	Model 2 β (95% CI) ***P*** value	Model 3 β (95% CI) ***P*** value
Dietary cholesterol (mg/day)	0.001 (0.000, 0.001) < 0.00001	− 0.000 (− 0.000, 0.000) 0.61114	− 0.000 (− 0.000, 0.000) 0.74345
Dietary cholesterol categories			
Q1 (0 to 125 mg/day)	Reference	Reference	Reference
Q2 (126 to 228 mg/day)	0.112 (0.001, 0.222) 0.04709	0.076 (−0.021, 0.173) 0.12641	0.021 (−0.084, 0.126) 0.69498
Q3 (229 to 403 mg/day)	0.296 (0.189, 0.404) < 0.00001	0.056 (−0.040, 0.152) 0.25331	0.043 (− 0.065, 0.150) 0.43615
Q4 (404 to 2403 mg/day)	0.346 (0.237, 0.455) < 0.00001	− 0.025 (− 0.123, 0.073) 0.62304	−0.030 (− 0.145, 0.085) 0.60950
Subgroup analysis stratified by gender			
Male	−0.000 (− 0.000, 0.000) 0.95983	−0.000 (− 0.000, 0.000) 0.85729	−0.000 (− 0.000, 0.000) 0.58699
Female	− 0.000 (− 0.000, 0.000) 0.60326	−0.000 (− 0.000, 0.000) 0.67516	0.000 (− 0.000, 0.000) 0.72075
Subgroup analysis stratified by race/ethnicity			
Mexican American	−0.000 (− 0.001, 0.000) 0.22398	−0.001 (− 0.001, − 0.000) 0.00155	−0.001 (− 0.001, 0.000) 0.08501
Other Hispanic	0.000 (− 0.000, 0.001) 0.07886	−0.000 (− 0.001, 0.000) 0.38447	−0.000 (− 0.001, 0.000) 0.31399
Non-Hispanic White	0.001 (0.001, 0.001) < 0.00001	0.000 (− 0.000, 0.000) 0.46732	0.000 (− 0.000, 0.000) 0.64506
Non-Hispanic Black	0.000 (− 0.000, 0.001) 0.35017	− 0.000 (− 0.001, 0.000) 0.14433	−0.000 (− 0.000, 0.000) 0.74005
Other races	0.001 (0.001, 0.001) < 0.00001	0.000 (− 0.000, 0.001) 0.21980	0.000 (− 0.000, 0.001) 0.52821

Model 1: no covariates were adjusted. Model 2: age, gender, and race/ethnicity were adjusted. Model 3: age, gender, race/ethnicity, alcohol consumption, smoking behavior, education level, marital status, the ratio of family income to poverty, energy, minutes of sedentary activity, weight, height, body mass index, waist circumference, hip circumference, glycohemoglobin, fasting glucose, total cholesterol, triglyceride, high-density lipoprotein cholesterol, low-density lipoprotein cholesterol, systolic blood pressure, and diastolic blood pressure were adjusted. In the subgroup analysis stratified by gender and race/ethnicity; the model is not adjusted for gender and race/ethnicity, respectively

**Fig. 7:**
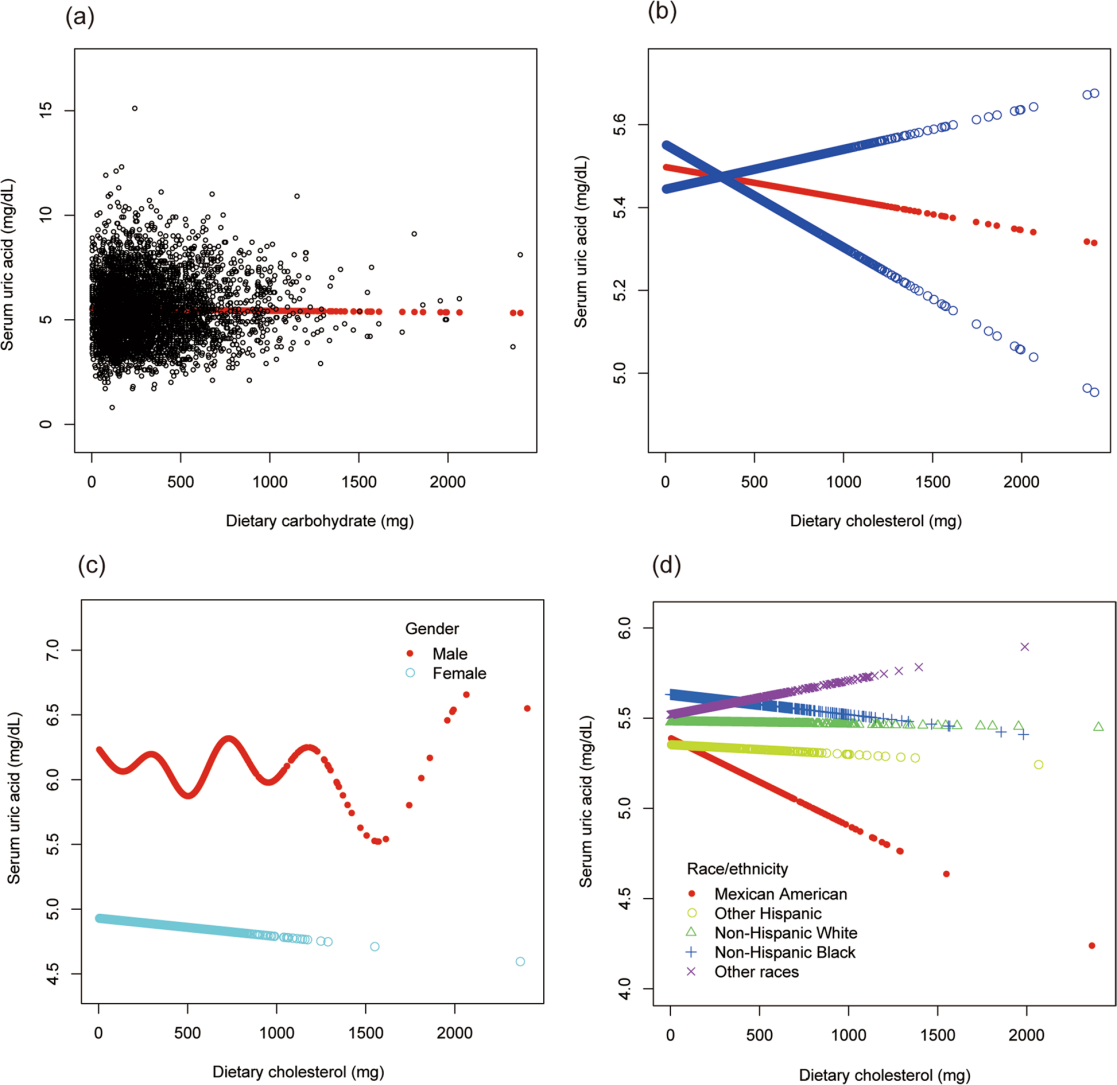
The association between dietary cholesterol and serum uric acid. **a** Each black point represents a sample. **b** The solid red line represents the smooth curve fit between variables. Blue bands represent the 95% of confidence interval from the fit. **c** Stratified by gender. **d** Stratified by race/ethnicity

To evaluate the nonlinear connection between dietary cholesterol intake and sUA stratified by gender and race/ethnicity, smooth curve fittings and generalized additive models were applied (Fig. 7).

#### Correlation of dietary fiber intake with sUA

As presented in Table 9, dietary fiber intake was negatively correlated with sUA (β = − 0.008, 95% CI: − 0.011 to − 0.004, *P* = 0.00001). Furthermore, individuals with higher dietary fiber intake (14.3 to 103.4 g/day) had lower sUA levels than those with lower dietary fiber intake (<= 9.0 g/day). According to the results of subgroup analyses stratified by gender and race, the negative association of dietary fiber intake with sUA remained in the subgroups of male (β = − 0.012, 95% CI: − 0.016 to − 0.007, *P* < 0.00001), Mexican American (β = − 0.013, 95% CI: − 0.021 to − 0.005, *P* = 0.00158), non-Hispanic White (β = − 0.008, 95% CI: − 0.015 to − 0.002, *P* = 0.00776), and other races (β = − 0.007, 95% CI: − 0.014 to − 0.000, *P* = 0.04040). According to the results of interaction analysis, the relationship between dietary fiber intake and sUA was significantly different in different gender (*P* interaction < 0.05) (Supplementary Table 1).

**Table 9:** The association between dietary fiber (g/day) and serum uric acid (mg/dL)

	Model 1 β (95% CI) ***P*** value	Model 2 β (95% CI) ***P*** value	Model 3 β (95% CI) ***P*** value
Dietary fiber (g/day)	0.000 (− 0.003, 0.004) 0.82106	− 0.011 (− 0.015, − 0.008) < 0.00001	− 0.008 (− 0.011, − 0.004) 0.00001
Dietary fiber categories			
Q1 (0.0 to 9.0 g/day)	Reference	Reference	Reference
Q2 (9.1 to 14.2 g/day)	− 0.030 (− 0.141, 0.080) 0.58817	− 0.020 (− 0.117, 0.076) 0.68143	0.001 (− 0.101, 0.103) 0.98865
Q3 (14.3 to 21.2 g/day)	− 0.012 (− 0.119, 0.096) 0.83102	−0.187 (− 0.282, − 0.092) 0.00012	−0.137 (− 0.238, − 0.037) 0.00724
Q4 (21.3 to 103.4 g/day)	−0.007 (− 0.117, 0.103) 0.90219	−0.284 (− 0.382, − 0.185) < 0.00001	−0.205 (− 0.310, − 0.100) 0.00013
Subgroup analysis stratified by gender			
Male	− 0.013 (− 0.017, − 0.008) < 0.00001	−0.014 (− 0.018, − 0.010) < 0.00001	−0.012 (− 0.016, − 0.007) < 0.00001
Female	−0.006 (− 0.012, − 0.001) 0.01594	−0.007 (− 0.012, − 0.002) 0.00570	−0.004 (− 0.009, 0.002) 0.18387
Subgroup analysis stratified by race/ethnicity			
Mexican American	−0.005 (− 0.013, 0.004) 0.30621	−0.016 (− 0.023, − 0.008) 0.00005	−0.013 (− 0.021, − 0.005) 0.00158
Other Hispanic	0.008 (− 0.003, 0.018) 0.14497	−0.008 (− 0.017, 0.000) 0.06390	−0.007 (− 0.017, 0.003) 0.15407
Non-Hispanic White	− 0.000 (− 0.007, 0.006) 0.97294	−0.012 (− 0.017, − 0.006) 0.00009	−0.008 (− 0.015, − 0.002) 0.00776
Non-Hispanic Black	0.004 (− 0.006, 0.013) 0.44917	−0.008 (− 0.016, 0.001) 0.07273	−0.006 (− 0.014, 0.003) 0.21369
Other races	0.003 (− 0.005, 0.010) 0.48494	−0.011 (− 0.017, − 0.005) 0.00066	−0.007 (− 0.014, − 0.000) 0.04040

Model 1: no covariates were adjusted. Model 2: age, gender, and race/ethnicity were adjusted. Model 3: age, gender, race/ethnicity, alcohol consumption, smoking behavior, education level, marital status, the ratio of family income to poverty, minutes of sedentary activity, weight, height, body mass index, waist circumference, hip circumference, glycohemoglobin, fasting glucose, total cholesterol, triglyceride, high-density lipoprotein cholesterol, low-density lipoprotein cholesterol, systolic blood pressure, and diastolic blood pressure were adjusted. In the subgroup analysis stratified by gender and race/ethnicity; the model is not adjusted for gender and race/ethnicity, respectively

**Fig. 8:**
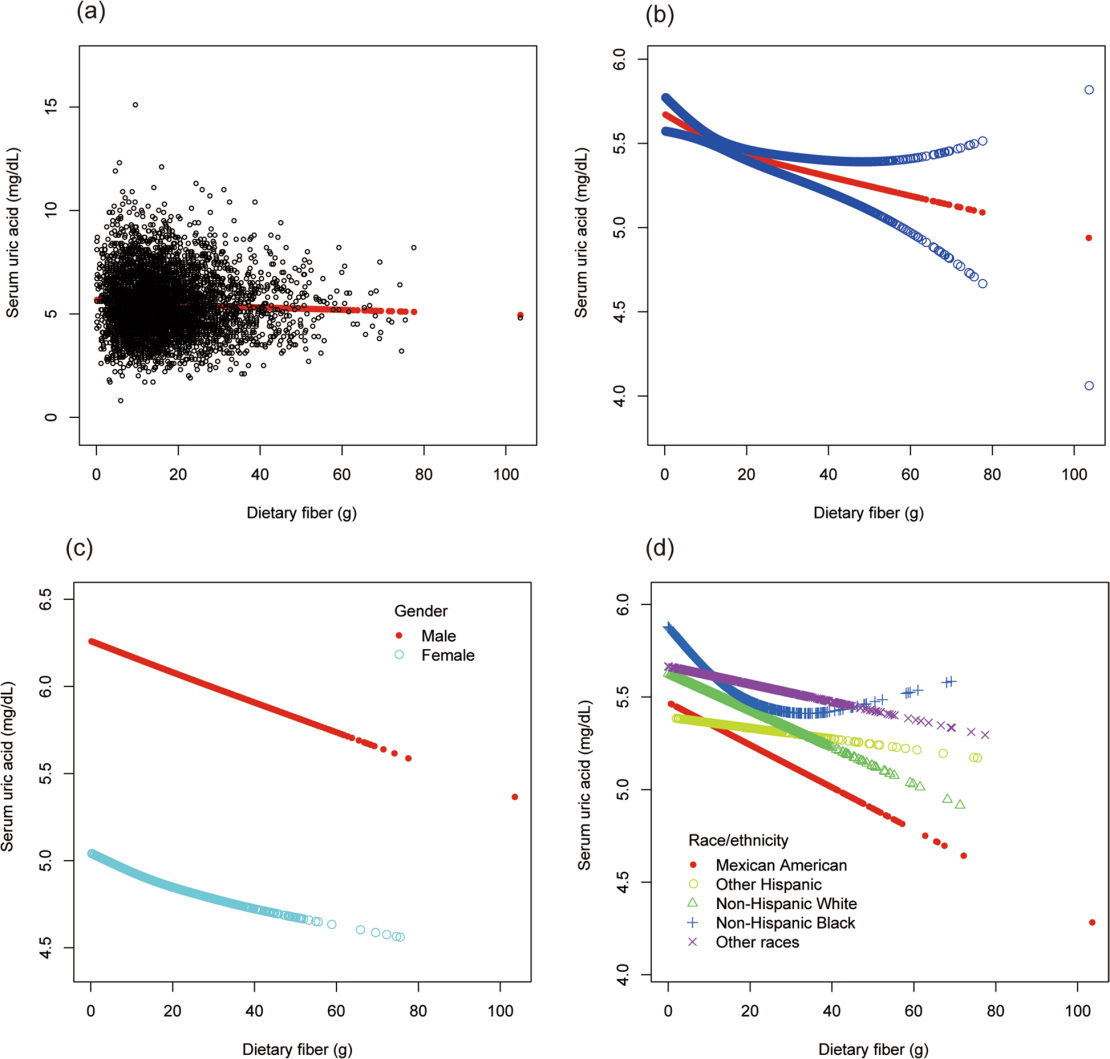
The association between dietary fiber and serum uric acid. **a** Each black point represents a sample. **b** The solid red line represents the smooth curve fit between variables. Blue bands represent the 95% of confidence interval from the fit. **c** Stratified by gender. **d** Stratified by race/ethnicity

To evaluate the nonlinear connection between dietary fiber intake and sUA stratified by gender and race/ethnicity, smooth curve fittings and generalized additive models were applied (Fig. 8).

### Correlation of BP with sUA

As shown in Table 10, systolic BP was positively correlated with sUA (β = 0.006, 95% CI: 0.003 to 0.009, *P* = 0.00002). Furthermore, individuals with higher systolic BP (120 to 140 mmHg or > = 140 mmHg) had higher sUA levels than those with the normal systolic BP (β = 0.194, 95% CI: 0.104 to 0.283, P = 0.00002; β = 0.167, 95% CI: 0.036 to 0.297, *P* = 0.01220; respectively). According to the results of subgroup analyses stratified by gender and race, the positive association of systolic BP with sUA remained in both male (β = 0.003, 95% CI: 0.000 to 0.007, *P* = 0.00844) and female (β = 0.004, 95% CI: 0.001 to 0.007, *P* = 0.00935), as well as in the race of non-Hispanic Black (β = 0.008, 95% CI: 0.003 to 0.013, *P* = 0.00340) and other races (β = 0.008, 95% CI: 0.003 to 0.013, *P* = 0.00277).

**Table 10:** The association between systolic blood pressure (mmHg) and serum uric acid (mg/dL)

	Model 1 β (95% CI) ***P*** value	Model 2 β (95% CI) ***P*** value	Model 3 β (95% CI) ***P*** value
SBP (mmHg)	0.014 (0.012, 0.016) < 0.00001	0.007 (0.005, 0.009) < 0.00001	0.006 (0.003, 0.009) 0.00002
SBP categories			
< = 120 mmHg	Reference	Reference	Reference
120 to140 mmHg	0.519 (0.436, 0.603) < 0.00001	0.238 (0.157, 0.318) < 0.00001	0.194 (0.104, 0.283) 0.00002
> = 140 mmHg	0.476 (0.362, 0.590) < 0.00001	0.190 (0.075, 0.305) 0.00126	0.167 (0.036, 0.297) 0.01220
Subgroup analysis stratified by gender			
Male	0.007 (0.004, 0.011) < 0.00001	0.005 (0.002, 0.009) 0.00362	0.003 (0.000, 0.007) 0.00844
Female	0.014 (0.012, 0.016) < 0.00001	0.007 (0.004, 0.010) 0.00002	0.004 (0.001, 0.007) 0.00935
Subgroup analysis stratified by race/ethnicity			
Mexican American	0.017 (0.011, 0.023) < 0.00001	0.010 (0.004, 0.017) 0.00165	0.004 (− 0.002, 0.011) 0.20657
Other Hispanic	0.020 (0.013, 0.026) < 0.00001	0.008 (0.001, 0.014) 0.02757	0.007 (−0.000, 0.015) 0.05725
Non-Hispanic White	0.012 (0.008, 0.016) < 0.00001	0.005 (0.001, 0.008) 0.02139	0.003 (−0.001, 0.007) 0.13653
Non-Hispanic Black	0.021 (0.016, 0.025) < 0.00001	0.011 (0.006, 0.016) 0.00001	0.008 (0.003, 0.013) 0.00340
Other races	0.012 (0.007, 0.017) < 0.00001	0.008 (0.003, 0.013) 0.00202	0.008 (0.003, 0.013) 0.00277

Model 1: no covariates were adjusted. Model 2: age, gender, and race/ethnicity were adjusted. Model 3: age, gender, race/ethnicity, alcohol consumption, smoking behavior, education level, marital status, the ratio of family income to poverty, energy, minutes of sedentary activity, glycohemoglobin, fasting glucose, total cholesterol, triglyceride, high-density lipoprotein cholesterol, low-density lipoprotein cholesterol, and diastolic blood pressure were adjusted. In the subgroup analysis stratified by gender and race/ethnicity; the model is not adjusted for gender and race/ethnicity, respectively

However, as showed in Table 11, no statistical difference was found in the associations of diastolic BP with sUA (β = − 0.002, 95% CI: − 0.005 to 0.001, *P* = 0.26232). Furthermore, no statistical difference was found in the subgroup analysis.

**Table 11:** The association between diastolic blood pressure (mmHg) and serum uric acid (mg/dL)

	Model 1 β (95% CI) ***P*** value	Model 2 β (95% CI) ***P*** value	Model 3 β (95% CI) ***P*** value
DBP (mmHg)	0.012 (0.009, 0.015) < 0.00001	0.003 (0.000, 0.006) 0.02054	−0.002 (− 0.005, 0.001) 0.26232
DBP categories			
< = 80 mmHg	Reference	Reference	Reference
81–89 mmHg	0.195 (0.091, 0.299) 0.00025	−0.001 (− 0.094, 0.092) 0.98499	−0.084 (− 0.187, 0.019) 0.10826
> = 90 mmHg	0.568 (0.413, 0.723) < 0.00001	0.205 (0.067, 0.344) 0.00374	0.031 (− 0.127, 0.189) 0.70058
Subgroup analysis stratified by gender			
Male	0.009 (0.005, 0.013) 0.00001	0.007 (0.003, 0.011) 0.00032	0.001 (−0.004, 0.006) 0.73198
Female	0.003 (−0.000, 0.007) 0.08736	−0.000 (− 0.004, 0.003) 0.85843	−0.004 (− 0.008, 0.001) 0.09175
Subgroup analysis stratified by race/ethnicity			
Mexican American	0.016 (0.009, 0.024) 0.00002	0.006 (−0.000, 0.013) 0.06241	−0.003 (− 0.011, 0.005) 0.50551
Other Hispanic	0.024 (0.014, 0.034) < 0.00001	0.007 (− 0.002, 0.016) 0.12152	0.003 (− 0.010, 0.016) 0.66079
Non-Hispanic White	0.009 (0.003, 0.014) 0.00099	.000 (−0.004, 0.005) 0.83021	− 0.004 (− 0.009, 0.002) 0.21863
Non-Hispanic Black	0.017 (0.010, 0.023) < 0.00001	0.005 (− 0.001, 0.012) 0.08842	− 0.003 (− 0.011, 0.005) 0.50348
Other races	0.014 (0.007, 0.020) 0.00003	0.008 (0.002, 0.013) 0.01117	0.001 (− 0.006, 0.008) 0.80664

Model 1: no covariates were adjusted. Model 2: age, gender, and race/ethnicity were adjusted. Model 3: age, gender, race/ethnicity, alcohol consumption, smoking behavior, education level, marital status, the ratio of family income to poverty, energy, minutes of sedentary activity, glycohemoglobin, fasting glucose, total cholesterol, triglyceride, high-density lipoprotein cholesterol, low-density lipoprotein cholesterol, and systolic blood pressure were adjusted. In the subgroup analysis stratified by gender and race/ethnicity; the model is not adjusted for gender and race/ethnicity, respectively

**Fig. 9:**
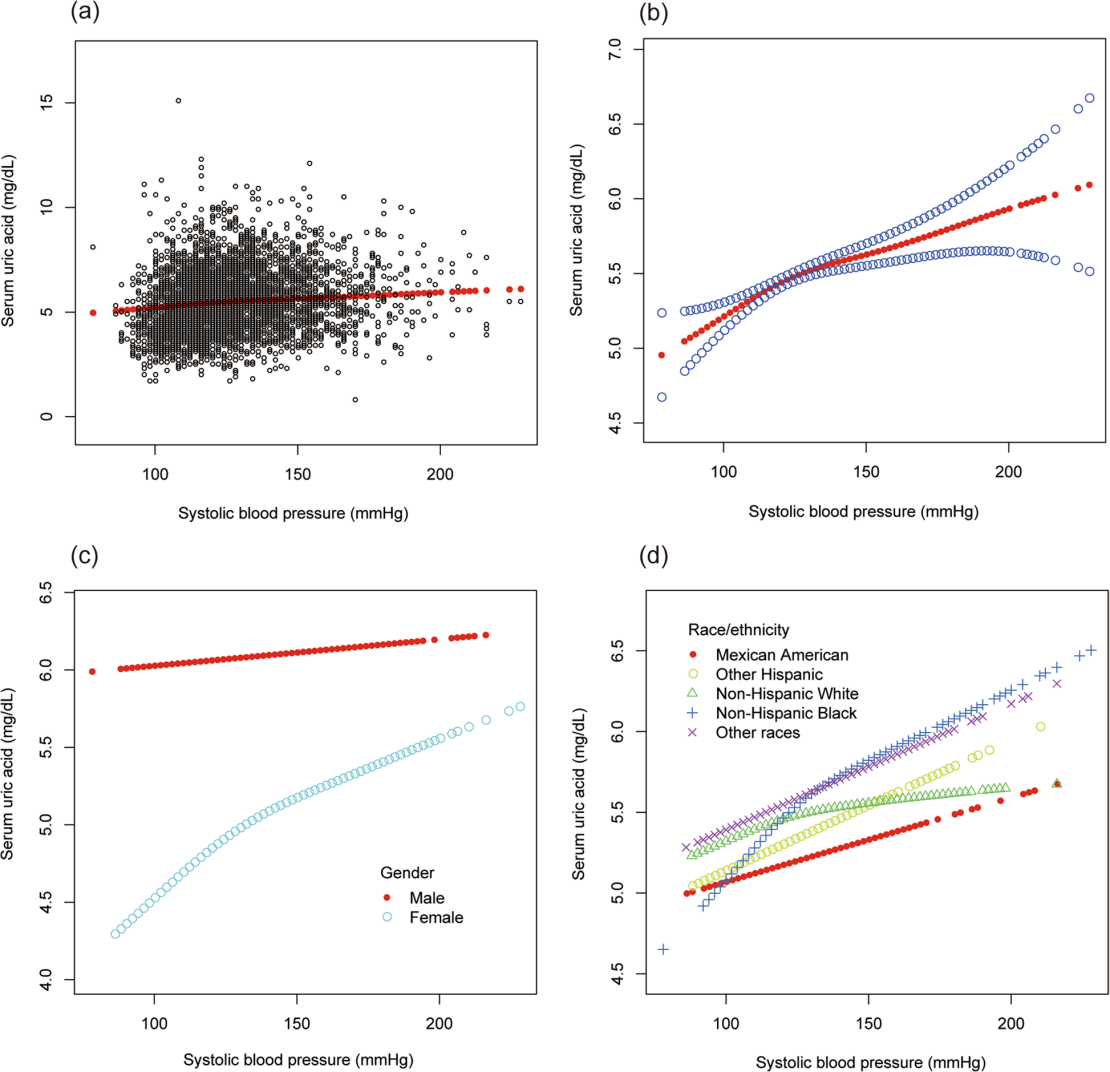
The association between systolic blood pressure and serum uric acid. **a** Each black point represents a sample. **b** The solid red line represents the smooth curve fit between variables. Blue bands represent the 95% of confidence interval from the fit. **c** Stratified by gender. **d** Stratified by race/ethnicity

**Fig. 10:**
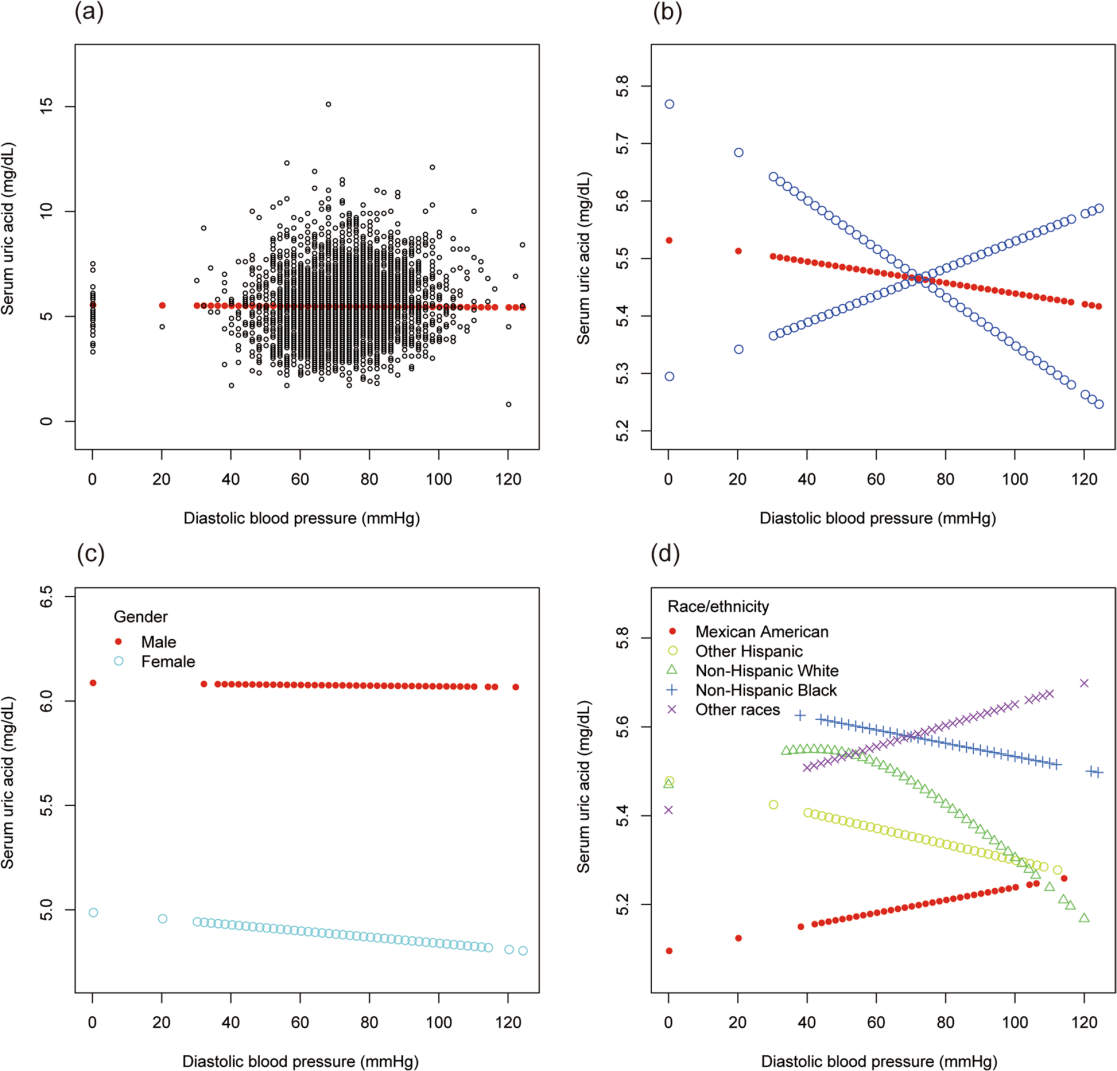
The association between diastolic blood pressure and serum uric acid. **a** Each black point represents a sample. **b** The solid red line represents the smooth curve fit between variables. Blue bands represent the 95% of confidence interval from the fit. **c** Stratified by gender. **d** Stratified by race/ethnicity

To evaluate the nonlinear connection of systolic and diastolic BP with sUA stratified by gender and race/ethnicity, smooth curve fittings and generalized additive models were applied (Figs. 9 and 10).

## Discussion

The current cross-sectional investigation of a nationally representative sample of US participants was conducted to investigate the association of obesity, dietary patterns, and BP with sUA. Results showed that BMI, dietary energy intake, and systolic BP were positively correlated with sUA levels. Furthermore, dietary carbohydrate and fiber intake were negatively correlated with sUA levels. However, no statistical difference was found in the associations of dietary intake of total sugars, protein, total fat, cholesterol, and diastolic BP with sUA levels.

According to our current findings, BMI was positively correlated with sUA levels, and this positive association was present in any gender and ethnic subgroup. This is consistent with earlier epidemiological and clinical evidence of a substantial significant positive connection between obesity and sUA in Chinese, Japanese, Indian, Pakistani, Iraqi, and Bangladeshi populations [2–4]. The link between obesity and sUA can be explained by several mechanisms: (1) increased liver synthesis: obese patients eat more calories than they consume, resulting in increased liver synthesis. Purine synthesis becomes hyperactive and UA production increases as energy intake rises. Lipolysis’ acidic metabolites can block UA excretion while indirectly increasing sUA levels. An increase in visceral fat raises the level of free fatty acids in the portal system. Furthermore, the liver’s fatty acid synthesis and the PRPP pathway are both overactive, resulting in enhanced triglyceride synthesis and UA generation [10]. (2) insulin resistance: obesity or extra body fat may be linked with elevated sUA production and insufficient excretion due to insulin resistance, resulting in impaired UA metabolism and even hyperuricemia [11]. Additionally, in the state of insulin resistance, the body can activate the renin-angiotensin-aldosterone system, causing a decrease in renal blood flow, resulting in a decrease in UA excretion and an increase in the sUA [12, 13]. (3) endocrine role of adipokines: a variety of adipokines (such as adiponectin, leptin, etc.) secreted by adipocytes play an important role in the pathogenesis of obesity, and HUA is closely related to and interacts with obesity. Therefore, these adipokines play an important role in HUA [5, 14]. (4) genetic factors: genetic factors may also play an important role, but the specific inheritance method is not clear, and it may be caused by genetic metabolic defects, that is, lipid metabolism disorders are accompanied by purine metabolism disorders. As a result of the intimate biological association between obesity and sUA, it is critical for preventive medicine to closely evaluate the interplay between sUA and obesity. Furthermore, BMI control is helpful for the management and treatment of hyperuricemia and gout.

Furthermore, sUA levels are greatly influenced by dietary factors. Our results showed that dietary energy intake was positively correlated with sUA levels. Increased energy intake was associated with increased purine synthesis and increased UA production. Therefore, we suggest that total energy intake should be limited in HUA patients. Furthermore, dietary carbohydrate and fiber intake were negatively correlated with sUA levels. Consistent with our findings, a study of American adults showed that higher total dietary fiber intake was associated with a lower incidence of HUA [15]. According to the results of interaction analysis, the relationship of dietary fiber intake with sUA was significantly different in different gender. Dietary fiber intake was negatively correlated with sUA in males instead of females. This difference may be related to the difference in the need for fiber intake and recommendation levels in different gender (12 g to 38 g in the male and 9.9 g to 25 g in the female). More research is needed to investigate the gender difference in the relationship of dietary fiber intake with sUA. As for the mechanism, the viscosity and volume of dietary fiber will hinder the absorption of purines by the digestive system, and dietary fiber delays the digestion process of fructose and slows down the rate of reabsorption in the small intestine. In addition, dietary fiber can aid free bowel movement and promote the excretion of UA in the intestine [15–17]. However, no statistical difference was found in the associations of dietary intake of total sugars with sUA levels in our study. At present, there is limited data about the influence of dietary total sugars intake on sUA. However, there are many studies on the correlation between fructose and sUA [18]. Research showed that dietary fructose was positively correlated with sUA levels [5]. Moreover, the long-term intake of fructose can also inhibit the excretion of UA by the kidneys and ileum, increasing sUA concentrations [19]. Our study only investigated the connection between dietary total sugars intake and sUA. There are many types of sugars, more research is needed to further investigate the correlation between different types of sugars and sUA. Additionally, the relationship between dietary total sugars intake and sUA was significantly different in the non-Hispanic White subgroup (P interaction < 0.05). Therefore, we speculate that this effect may have racial differences. More research is needed to investigate the racial difference in the relationship between dietary total sugars intake and sUA. Similar to our findings, several studies also found that the total protein intake was not associated with sUA level [20–23]. However, in the subgroup analysis, we found that dietary protein intake was negatively correlated with sUA in male, Mexican American, and other Hispanic subjects. Thus, we speculate that there may be gender and ethnic differences in the correlation of dietary protein intake with sUA. More investigations on the physiology of gender and ethnic differences are needed. Furthermore, no statistical difference was found in the associations of dietary total fat and cholesterol intake with sUA. However, individuals with the highest dietary total fat intake (108.11 to 576.96 g/day) had higher sUA levels than those with lower dietary total fat intake (<= 50.74 g/day). A mechanism linking fat intake and uric acid metabolism may involve the effects of dietary fats on insulin sensitivity [24]. Thus, dietary total fat intake may also be restricted for HUA and gout patients.

Our findings further indicated that systolic BP was positively correlated with sUA. And individuals with higher systolic BP (> 120 mmHg) had higher sUA levels than those with normal systolic BP. However, no statistical difference was found in the associations of diastolic BP with sUA in the present study. Epidemiological, clinical, and experimental evidence supports that the elevated UA level can increase the risk of arterial hypertension [25]. However, there are few studies investigating the effects of elevated BP on sUA. Hypertension itself may not lead to increased UA, but the long-term existence of elevated BP may lead to hypertensive nephropathy, which will affect the excretion of UA, thus leading to HUA. Furthermore, HUA can further increase BP by the deleterious effects on the kidney, the activation of the intrarenal renin-angiotensin system, the deposition of urate crystals in the urinary lumen, and direct endothelial injury [25]. For the intimate biological association between BP and sUA, it is critical to block the vicious circle of hypertension-HUA. Clinically, BP control may be also helpful for the management and treatment of hyperuricemia and gout.

The current study’s limitations stem mostly from its cross-sectional methodology, which cannot support a causal association of obesity, dietary patterns, and BP with sUA. Secondly, because the NHANES is a sample survey, generalizability may be hampered by selection bias. As a result, further fundamental mechanistic investigations and large-sample prospective research are needed to determine the precise mechanism of the relationship between obesity, dietary patterns, and BP with sUA. Thirdly, we were unable to obtain more detailed data since this survey did not include questions concerning gout diagnosis or medication use, such as urate-lowering drugs and other medicines that might alter sUA levels, body weight, and BP. Fourthly, the risk of bias due to other potential variables that we could not account for remains. Despite its limitations, the findings might be helpful for the management and treatment of HUA and gout because of the positive correlation of BMI, dietary energy intake, and systolic BP with sUA levels, and the negative correlation of dietary carbohydrate and fiber intake with sUA levels.

## Conclusion

The current cross-sectional investigation of a nationally representative sample of US participants showed that BMI, dietary energy intake, and systolic BP were positively correlated with sUA levels; dietary carbohydrate and fiber intake were negatively correlated with sUA levels. The findings might be helpful for the management and treatment of HUA and gout.

## Supplementary Information

**Additional file 1: Supplementary Table 1.** Results of interaction analysis.

## Data Availability

The complete data can be found at http://www.cdc.gov/nchs/nhanes/. And the data used to support the findings of this study are available from corresponding author upon reasonable request.

## Abbreviations

UA: Uric acid
HUA: Hyperuricemia
US: United States
BP: Blood pressure
sUA: Serum uric acid
NHANES: National Health and Nutrition Examination Survey
BMI: Body mass index
