# Supplementary material for: Correlation of obesity, dietary patterns, and blood pressure with uric acid: data from the NHANES 2017–2018
Source: BMC Endocr Disord. 2022 Aug 5;22:196. doi: 10.1186/s12902-022-01112-5 (PMC9354319; doi:10.1186/s12902-022-01112-5)
Supplement: Supplementary file 1 — Additional file 1: Supplementary Table 1. Results of interaction analysis. [file 12902_2022_1112_MOESM1_ESM.docx]

**Supplementary Table 1. Results of interaction analysis.**

| **Outcomes** | **Gender** | **Race/ethnicity (Mexican American as reference)** | | | |
| --- | --- | --- | --- | --- | --- |
|  |  | **Other Hispanic** | **Non-Hispanic White** | **Non-Hispanic Black** | **Other races** |
| Body mass index (kg/m^2^) | 0.001 (-0.013, 0.015)  >0.05 insignificant | 0.004 (-0.003, 0.011)  >0.05 insignificant | 0.001 (-0.009, 0.011)  >0.05 insignificant | 0.004 (-0.011, 0.018)  >0.05 insignificant | 0.007 (-0.013, 0.026)  >0.05 insignificant |
| Dietary energy | 0.001 (-0.000, 0.001)  >0.05 insignificant | 0.000 (-0.000, 0.000)  >0.05 insignificant | -0.000 (-0.000, 0.000)  >0.05 insignificant | -0.000 (-0.000, 0.000)  >0.05 insignificant | -0.000 (-0.000, 0.000)  >0.05 insignificant |
| Dietary carbohydrate | -0.000 (-0.001, 0.000)  >0.05 insignificant | 0.000 (-0.001, 0.001)  >0.05 insignificant | -0.001 (-0.001, 0.000)  >0.05 insignificant | -0.001 (-0.002, 0.000)  >0.05 insignificant | -0.001 (-0.002, 0.001)  >0.05 insignificant |
| Dietary sugars | 0.000 (-0.001, 0.002)  >0.05 insignificant | -0.000 (-0.002, 0.001)  >0.05 insignificant | -0.002 (-0.004, -0.000) <0.05 | -0.002 (-0.004, 0.000)  >0.05 insignificant | -0.002 (-0.005, 0.000)  >0.05 insignificant |
| Dietary protein | 0.001 (-0.002, 0.003)  >0.05 insignificant | 0.001 (-0.001, 0.003)  >0.05 insignificant | 0.000 (-0.002, 0.002)  >0.05 insignificant | 0.001 (-0.002, 0.004)  >0.05 insignificant | 0.002 (-0.002, 0.006)  >0.05 insignificant |
| Dietary total fat | 0.001 (-0.001, 0.003)  >0.05 insignificant | 0.001 (-0.001, 0.0003)  >0.05 insignificant | -0.000 (-0.002, 0.001)  >0.05 insignificant | -0.001 (-0.003, 0.001)  >0.05 insignificant | 0.000 (-0.003, 0.003)  >0.05 insignificant |
| Dietary cholesterol | 0.000 (-0.000, 0.000)  >0.05 insignificant | 0.000 (-0.000, 0.001)  >0.05 insignificant | 0.000 (-0.000, 0.001)  >0.05 insignificant | 0.000 (-0.000, 0.001)  >0.05 insignificant | 0.001 (-0.000, 0.001)  >0.05 insignificant |
| Dietary fiber | -0.007 (-0.014, -0.000) <0.05 | 0.006 (-0.002, 0.014)  >0.05 insignificant | -0.002 (-0.009, 0.006)  >0.05 insignificant | 0.002 (-0.008, 0.013)  >0.05 insignificant | 0.002 (-0.012, 0.016)  >0.05 insignificant |
| Systolic blood pressure | -0.007 (-0.012, 0.001)  >0.05 insignificant | 0.001 (-0.001, 0.003)  >0.05 insignificant | 0.001 (-0.003, 0.005)  >0.05 insignificant | 0.003 (-0.003, 0.009)  >0.05 insignificant | 0.003 (-0.005, 0.011)  >0.05 insignificant |
| Diastolic blood pressure | 0.004 (-0.004, 0.013)  >0.05 insignificant | 0.001 (-0.003, 0.004)  >0.05 insignificant | -0.000 (-0.006, 0.006)  >0.05 insignificant | 0.002 (-0.006, 0.010)  >0.05 insignificant | 0.001 (-0.010, 0.012)  >0.05 insignificant |
